# Role of family medicine physicians in providing nutrition support to older patients admitted to orthopedics departments: a grounded theory approach

**DOI:** 10.1186/s12875-024-02379-4

**Published:** 2024-04-19

**Authors:** Ryuichi Ohta, Tachiko Nitta, Akiko Shimizu, Chiaki Sano

**Affiliations:** 1 Community Care, Unnan City Hospital, 96-1 Iida, Daito-cho, Unnan, Shimane, 699-1221 Japan; 2 Department of Community Medicine Management, Faculty of Medicine, Shimane University, 89-1 Enya cho, Izumo, Shimane, 693-8501 Japan

**Keywords:** Nutrition support team, Family medicine, Orthopedic conditions, Rural, Community, Hospital, Interprofessional collaboration, Japan

## Abstract

**Background:**

Care of older adults requires comprehensive management and control of systemic diseases, which can be effectively managed by family physicians. Complicated medical conditions in older patients admitted to orthopedic departments (orthopedic patients) necessitate interprofessional collaboration. Nutrition is one of the essential components of management involved in improving the systemic condition of older patients. Nutrition support teams play an important role in nutrition management and can be supported by family physicians. However, the role of family physicians in nutrition support teams is not well documented. This study aimed to investigate the role of family physicians in supporting nutrition management in orthopedic patients.

**Methods:**

This qualitative study was conducted between January and June 2023 using constructivist grounded theory methodology. Eight family medicine physicians, three orthopedic surgeons, two nurses, two pharmacists, four rehabilitation therapists, four nutritionists, and one laboratory technician working in Japanese rural hospitals participated in the research. Data collection was performed through ethnography and semi-structured interviews. The analysis was performed iteratively during the study.

**Results:**

Using a grounded theory approach, four theories were developed regarding family physicians’ role in providing nutrition support to orthopedic patients: hierarchical and relational limitation, delay of onset and detection of the need for geriatric care in orthopedic patients, providing effective family medicine in hospitals, and comprehensive management through the nutrition support team.

**Conclusions:**

The inclusion of family physicians in nutrition support teams can help with early detection of the rapid deterioration of orthopedic patients’ conditions, and comprehensive management can be provided by nutrition support teams. In rural primary care settings, family physicians play a vital role in providing geriatric care in community hospitals in collaboration with specialists. Family medicine in hospitals should be investigated in other settings for better geriatric care and to drive mutual learning among healthcare professionals.

**Supplementary Information:**

The online version contains supplementary material available at 10.1186/s12875-024-02379-4.

## Background

Care of older adults requires comprehensive management and control of systemic diseases, which family physicians can manage efficiently [1, 2]. Specialists can provide effective management of specific diseases for young and middle-aged patients. However, older patients often have complicated medical conditions with multiple comorbidities; therefore, specialists may not be equipped to manage older patients’ healthcare needs related to treatment for other specific conditions and chronic diseases [3, 4]. Family physicians are generalists dealing with multimorbidity according to the biopsychosocial model [5]. Family physicians can systematically control the range of health problems in patients admitted to support specialists’ treatments. Thus, in aging societies, the collaboration between specialists with system-specific and multisystem expertise is vital for the comprehensive care of older patients.

The increase in the number of older patients admitted to orthopedic departments (orthopedic patients) complicates hospital care and necessitates interprofessional collaboration. Older orthopedic patients with fractures and other musculoskeletal damage need interventions such as surgeries and multiple medicines to reduce pain and improve their activities of daily living (ADL) [6, 7]. However, older patients, are more likely to develop complications and exacerbations owing to their chronic health conditions. Orthopedic surgeons collaborate with family physicians in hospital care to manage complicated medical conditions in older patients [8]. To effectively manage older hospitalized patients, family physicians can coordinate comprehensive care, including medical care, rehabilitation, and nutrition.

Nutrition support teams play an important role in nutrition management among older orthopedic patients and can be supported by family physicians [9, 10].Currently, hospital care is provided by specialists, but demand for comprehensive care has been increasing worldwide, especially in the realms of nutrition and rehabilitation [9, 10]. Rehabilitation and nutrition support are essential for helping patients regain their ADL [10]. Nutrition support teams in hospitals are essential for managing systemic conditions in older patients [11]. Nutritional conditions can be affected by subtle changes in systemic conditions [12]. Discussion among members of nutrition support teams, including family physicians and specialists, can detect early changes in the medical condition of hospitalized patients and prevent the development of complications [13].

Nutrition support teams consist of multiple healthcare professionals, such as physicians, nutritionists, therapists, nurses, and pharmacists [14]. The effectiveness of nutrition support teams can be affected by the quality of interaction among team members in making decisions regarding nutrition management [14]. Family physician involvement can improve the quality of the discussion and management of patients requiring care by specialist physicians, because the specialty of family medicine is adept at managing multimorbidity and using a biopsychosocial approach [15]. However, the effectiveness of family physician involvement in nutrition support teams and its effect on patient management is not well documented. This study aimed to investigate family physicians’ role in providing nutrition support to older patients with orthopedic conditions as part of a multidisciplinary nutrition support team.

## Methods

This qualitative study was conducted from January to June 2023, based on the methodology of constructivist grounded theory.

### Setting

Unnan City is one of Japan’s smallest and most remote cities and is located southeast of an administrative unit in a rural setting. In 2020, the city’s total population was 37,638 (18,145 males and 19,492 females), and 39% of the population was aged over 65 years. This proportion is expected to reach 50% by 2025. The city has 16 clinics, 12 home care stations, 3 visiting nurse stations, and 1 public hospital. At the time of the study, Unnan City Hospital had 281 care beds, including 160 acute care, 43 comprehensive care, 30 rehabilitation, and 48 chronic care beds. The nurse-to-patient ratios were 1:10, 1:13, 1:15, and 1:25 for acute care, comprehensive care, rehabilitation, and chronic care, respectively. The hospital had 27 physicians, 1 dentist, 197 nurses, 7 pharmacists, 15 clinical technicians, 37 therapists, 4 nutritionists, and 34 clerks [16].

Unnan City Hospital has a Family Medicine Department, which cares mainly for internal medicine inpatients and outpatients. The members of the Family Medicine Department comprise family medicine specialists and trainees in family medicine. The department trains medical residents using an educational curriculum based on the Japanese Primary Care Association’s Board of Family Medicine, developed according to the World Standard of Education of Family Medicine. The department can train maximum of three residents simultaneously. During the study period, the hospital had three medical educators specialized in family medicine. The department trained one resident each in 2018 and 2019 and three residents each in 2020, 2021, and 2022 in the curriculum. In 2022 and 2023, these 10 physicians (2 family physicians and 8 family medicine residents) were part of the Family Medicine Department. This department promotes interprofessional collaboration with the hospital’s dentists, pharmacists, therapists, nurses, and nutritionists. Nutritionists are officially certified by the Japanese government. Ongoing interprofessional collaboration in managing older inpatients has reduced the readmission rate [16].

### Collaboration in the nutrition support team

The nutrition support team consists of family physicians, nutritionists, nurses, and pharmacists. Provision of nutrition support starts with asking nurses working in each hospital ward to assess and refer patients with undernutrition. Orthopedic patients are screened for nutritional conditions on admission, and family physicians are consulted regarding the diet of patients with undernutrition based on their observed food intake and changes in their nutritional condition based on laboratory nutritional assessment [16].

The nutrition support team has meetings once a week. The nutritional condition of inpatients is assessed using a mini-nutritional assessment tool, which is generally used in Japan to assess nutritional conditions using a simple questionnaire. Nutritionists consult family physicians, provide meal plans with appropriate nutritional components, and monitor the food intake of patients assessed as undernourished. Nurses provide input on patients’ conditions during team discussions from a care perspective. Pharmacists assess the medication conditions of patients and identify unnecessary medications based on the patient’s clinical condition in consultation with family physicians.

The nutritional condition of inpatients is assessed based on laboratory changes, such as lymphocyte count, serum albumin, and cholinesterase levels. Family physicians check the laboratory data of patients three days, one week, and two weeks after admission to assess their nutritional condition. If the laboratory markers of nutritional condition deteriorate, the nutritionists review the patients’ nutritional condition again. Through discussions with family physicians, nutritionists modify the meal form and content.

In this study, we focused on assessing the nutrition support teams’ comprehensive management of nutrition support for orthopedic patients. The nutrition support teams deal with patients from different medical departments. Orthopedic surgeons specialize in surgical procedures and need support to provide comprehensive management of older orthopedic patients with multimorbidity; therefore, through dialogue with orthopedic surgeons in the hospital, we offered to assist with comprehensive management of orthopedic patients and provide intensive nutrition support.

### Participants

All Family Medicine, Orthopedics, and Nutrition Department members participated in this research. All participants were informed about the research and provided written informed consent. The participants comprised 8 family medicine physicians, 3 orthopedic surgeons, two nurses, 2 pharmacists, 4 rehabilitation therapists, 4 nutritionists, and 1 laboratory technician. The family physicians had 4 to 12 years of clinical experience. The other participants had more than 10 years of clinical experience.

### Data collection

#### Ethnography

The study objectives were addressed using an ethnographic approach. Three researchers (RO, TN, and AS) acted as participatory investigators and worked as members of the nutrition support team. RO worked in all hospital wards and observed and interacted with all members of the Family Medicine, Orthopedics, and Nutrition Departments in each ward and during meetings, and took field notes of the observations. TN worked as a nutritionist and had discussions with the other nutritionists regarding the challenges that they faced in the care of orthopedic patients and the benefits of Family Medicine Department involvement. AS worked as a registered nurse and specialized in dysphagia in older patients. AS had discussions with the nurses and pharmacists in each ward about the challenges that they faced in the management of orthopedic patients and the benefits of Family Medicine Department involvement.

Once a week, RO, TN, and AS discussed the challenges and benefits of Family Medicine Department involvement. RO reviewed the field notes about the care of orthopedic patients from a family medicine perspective, and asked TN and AS whether they had had similar conversations with the other participants. RO, TN, and AS shared their perceptions regarding Family Medicine Department involvement in the care of orthopedic patients during these weekly discussions. Specifically, they reflected on their observations and tried to gain an in-depth understanding of one another’s perspective. The contents of the discussion were noted by RO for use in observation and to guide semi-structured interviews.

### Semi-structured interviews

During the study period, RO interviewed each participant in the hospital conference rooms about their perspectives regarding the care of specific patients after the patient’s discharge or death. The interview guide included three questions: (1) “*What did you think of the involvement of the Family Medicine Department with orthopedic patients through nutrition support teams?*” (2) “*What do you consider to be the challenges and benefits of* the *involvement of the Family Medicine Department in nutrition support teams?*” and (3) “*Do you have any suggestions on addressing the challenges and benefits of the involvement of the Family Medicine Department?*”(Appendix 1) During the interview, RO reviewed the field and discussion notes and inquired about the challenges and benefits of Family Medicine Department involvement. Each interview lasted 21–47 minutes and was recorded and transcribed verbatim. After each interview, RO, TN, and AS discussed the challenges and benefits of Family Medicine Department involvement.

### Analysis

This study used an inductive grounded theory approach [17]. After reading the contents of the field notes, conducting semi-structured in-depth interviews, and holding discussions with TN and AS, RO coded the content and developed codebooks based on repeated reading of the field notes to assess the initial coding for reliability (open coding). This study used process and concept coding methods [18]. RO, TN, and AS discussed the initial coding by reviewing the field notes and interview contents until they reached consensus. Further, RO induced, merged, deleted, and refined the coding, creating concepts by going back and forth between the research data and the initial coding for axial coding. The axial coding focused on grouping tentative concepts and creating tentative themes, accompanied by refining the codes, concepts, and themes. RO, TN, and AS discussed concepts and themes continuously for triangulation. To obtain theoretical saturation, the interview contents were analyzed iteratively during the research period after each participant completed competency-based medical education training. To reach consensus, the analysis result was provided to all participants, whose additional feedback and ideas were then included in the final revision of themes and concepts. Finally, the theory was discussed by all the research team members (RO, TN, and CS), and they agreed on the final themes.

### Reflexivity

The study results were co-created by the researchers and other participants through interactions. The research team members possessed diverse expertise and perspectives on rural medical education. RO, a family physician and medical teacher, graduated with a master’s degree in family medicine and public health and had experience working and researching in rural contexts. RO participated in this research representing the physician perspective. TN, a hospital nutritionist, had managed the Nutrition Department at Unnan City Hospital for 20 years. TN participated in this research representing the nutritionist perspective. AS, a registered nurse who had specialized in dysphagia management, had managed nutrition and dysphagia issues at Unnan City Hospital for 10 years. AS participated in this research representing the nursing perspective. CS, a medical educator and professor at a medical university, had specialized in community health care management and education. CS participated in this research representing the perspective of hospital managers. To prevent biases, the research team discussed the findings of individual data analyses and explored alternative viewpoints during the process of data interpretation.

### Ethical considerations

The Unnan City Hospital Clinical Ethics Committee approved the study protocol (approval number: 20,210,023).

## Results

### Findings based on a grounded theory approach

**Fig. 1:**
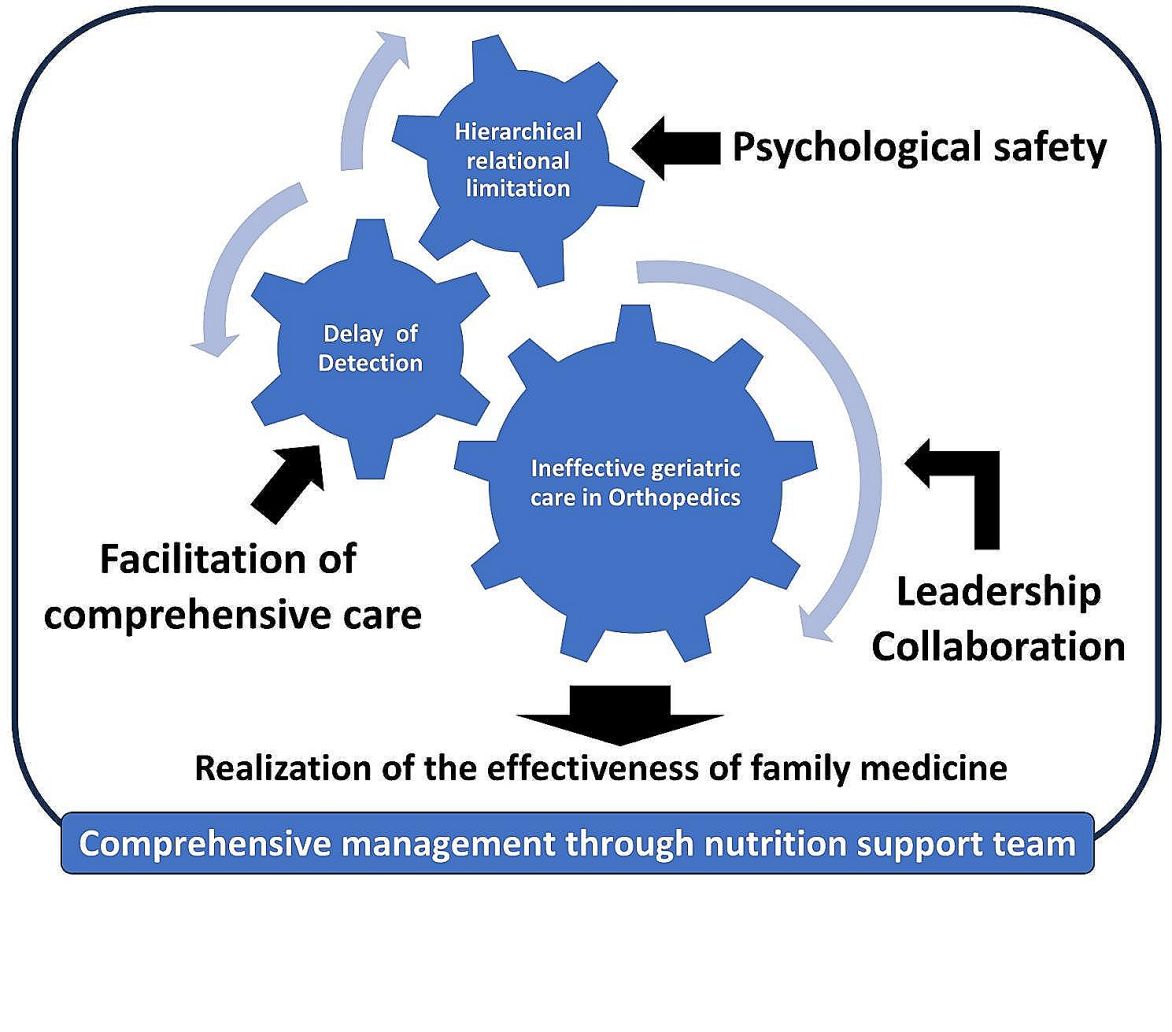
Conceptual diagram of family physicians’ role in providing nutrition support to older orthopedic patients

The family physicians had 4 to 12 years of clinical experience. The other participants had more than 10 years of clinical experience. Using a grounded theory approach, four themes were developed regarding family physicians’ role in providing nutrition support to orthopedic patients: (1) hierarchical and relational limitations; (2) delay and detection of geriatric care in orthopedic patients in need of nutrition support; (3) realization of the effectiveness of family medicine in hospitals; and (4) comprehensive management through the nutrition support team (Fig. 1).

Interpersonal relationships can hinder collaboration among healthcare professionals from different disciplines. Participants were intensely aware of their respective roles, which resulted in limited coordination. In particular, several participants felt a disparity between their role and that of physicians, and avoided expressing their opinions to physicians. As a result, there was an unconscious adherence to the professional hierarchy. Consequently, as experts, they felt increasingly frustrated about not being able to use their knowledge.

In an aging society, the changes in the condition of older patients have become more diverse and the need for medical care has become more frequent. Due to the nature of orthopedic surgery, the higher number of patients requiring surgery led to delays in responding to changes in the nutritional condition of orthopedic patients due to the heavy workload of orthopedic surgeons. Therefore, several orthopedic patients experienced a rapid deterioration in their condition during hospitalization. Older orthopedic patients are prone to malnutrition. Through the involvement of the nutrition support team, there was an increasing awareness of changes in patients’ nutritional status.

Including specialists in nutrition support teams promotes increasing recognition of other abnormalities in patients’ conditions. The presence of a family physician in the nutrition support team serves as a trigger for noticing various abnormalities. Furthermore, the comprehensive perspective and expertise of family physicians enables them to play an important role within the nutrition support team in facilitating consensus and the recognition of diverse patient needs.

The nutrition support team, including the family physician, recognized changes in patients’ overall symptoms effectively by identifying malnutrition in orthopedic patients. By actively seeking the opinions of various team members, with the family physician as a central figure, the nutrition support team intervened effectively by consolidating the perspectives of diverse specialists.

### Hierarchical and relational limitations

#### Consideration of interpersonal relationships

Consideration of interpersonal relationships is a hindrance to interprofessional collaboration. Traditionally, healthcare professionals are expected to perform their specified roles and not interfere with care provided by other healthcare professionals. Therefore, some participants stated that they tended to avoid reporting issues to other professionals, even when they noticed some drawbacks. Nutritionist A stated,

*I did not openly mention drawbacks to other professionals. Each professional may strongly feel that this is my work, and the other tasks are not*.

Thus, participants from each profession were intensely aware of their specific role, resulting in insufficient coordination. Some participants thought that they should collaborate with other professionals by sharing opinions openly, but the established work culture tended to inhibit proactive behavior. Nurse B stated,

*I did not act against the previous conception of doing only my jobs. I knew I should tell other professionals that they could revise their work for patients. but I don’t feel able to do so*.

### Disparity among healthcare professionals and persistent professional hierarchy

Several participants felt a disparity between their role and that of physicians and avoided expressing their opinions to physicians. The participants emphasized that there was a professional hierarchy between physicians and other healthcare professionals and that other professionals were hesitant to raise issues with physicians. Nutritionist B stated,

*I could not say anything to physicians. I am unsure and can be affected by professional education or clinical experience. I tend to hesitate to say something to physicians*.

Pharmacist B stated,

*Negative experiences might affect my attitude toward physicians. I was scolded and negated by physicians once. Physicians’ opinions are always strong, so I cannot say things to them*.

As a result of various negative experiences in education and clinical experience, there was an unconscious adherence to professional hierarchy. Consequently, as experts, other healthcare professionals increasingly felt frustrated about not being able to use their knowledge.

Nutritionist A stated,

*I could use my clinical knowledge with my patients, but the relationships with physicians might impinge on my behavior*.

Most participants believed that the professional hierarchy should be dispelled but stated that hierarchical relationships were persistent in interprofessional collaboration in the rural context.

### Delayed detection of undernutrition in older orthopedic patients

#### Changes in the clinical condition and nutrition status of older patients

In an aging society, the healthcare needs of older inpatients have become increasingly more frequent and diverse. Orthopedic surgeons stated that their specialty did not address aged-related changes in patients’ health conditions other than orthopedic diseases. Some participants found it challenging to manage older patients in the Orthopedic Department effectively.

Nurse C stated,

*I did not learn much about the chronic care of older patients except for orthopedic issues. Older patients show multiple changes after surgery, which may be affected by their multiple medical conditions and medicines. I am not confident in controlling their symptoms effectively*.

Other participants emphasized that the aging of the population increased the need for effective collaboration, especially in the management of conditions of specific organs. The lack of effective collaboration detracted from the quality of care received by older patients in a rural hospital setting.

Nutritionist C stated,

*The changes in older patients need comprehensive care. My specialty is nutrition, and I work on nutrition support. But older patients’ issues cannot be solved from one aspect alone. I have to ask for help from other professionals. Without the support of other professionals, I cannot work effectively to support patient care*.

The increase in the number of older patients demanded further interprofessional collaboration by sharing of patient information, but actual working situations did not fit the need for providing care to older patients.

### Delayed detection of acute changes in the nutritional condition of older patients

Due to the nature of orthopedic surgery, rural orthopedic surgeons had to perform surgery on many older patients, which was very time-consuming. Rural orthopedic surgeons did not have sufficient time to manage older patients comprehensively, which can cause delays in responding to changes in the condition of orthopedic patients. The orthopedic surgeons stated that they did not have enough time to care for subtle changes in older patients caused by the presence of multimorbidity and polypharmacy.

Orthopedic Surgeon A stated,

*I do not have time to take care of complications unrelated to surgery and the systemic symptoms caused by patients chronic diseases*.

Orthopedic Surgeon B stated,

“*I wanted to focus on orthopedic surgery, but changes in older patients’ clinical conditions are sometimes unpredictable. Their changes are stressful for me and delay our detection of them*.”

Other participants noticed a delay in detecting subtle changes in older patients. However, they could not bring the changes directly to the attention of physicians because of the professional hierarchy and their lack of confidence in providing medical care. Their hesitation and reluctance to speak up led to delays in addressing changes in the medical condition of older patients.

Nurse E stated,

“*I detected some subtle changes in older patients’ through the conversations and observing their medical charts. I knew that I should report the changes to the orthopedic surgeons soon, but I was not confident, and I felt the hierarchy with them. I could not tell the orthopedic surgeons, and this caused a deterioration in the patients’ symptoms*.”

### Delayed response to subtle changes in the nutritional condition of older patients

Many orthopedic patients’ experienced a rapid deterioration in their condition while hospitalized. Through the involvement of the nutrition support team, there was an increasing awareness of changes in patients’ nutritional condition. The detection of the changes among older patients was delayed because nutritional deterioration appeared after the progression of their clinical conditions. Some participants observed that patients’ clinical condition worsened when involvement of nutrition support team members was delayed.

Nutritionist D stated,

*Our nutritional approaches can be complicated because of the delay in the detection of acute conditions leading to poor nutritional conditions*.

Subtle changes could be challenging for orthopedic surgeons and nurses to detect because of their professional roles. There was general agreement among participants that early involvement of generalists is necessary for orthopedic patients with acute symptoms.

Family Physician B stated,

*Management of older patients in orthopedics needs comprehensive care by generalists such as family physicians. However, family medicine was not well known in my hospital, so the implementation of the specialty has also been delayed*.

### Effectiveness of family medicine in managing the nutrition support needs of older patients in a community hospital

#### Comprehensive assessment by nutrition support team

Through the approach of the nutrition support team to orthopedic patients, there has been increased recognition of abnormalities in patients’ conditions other than nutrition. The participants stated that nutrition support teams needed to provide comprehensive management of patients, including nutrition and management of other complications.

Nutritionist A stated,

*In the approach to older patients, the nutrition support team has to deal with multiple medical problems to solve nutritional issues*.

Comprehensive management could not be provided because of a lack of perspective regarding providing systemic care for older people. The nutrition support teams stated that the input of family physicians’ is required to provide nutrition support to older patients.

Family Physician A stated,

*Comprehensive management is demanded in nutrition support teams to deal with nutritional issues among older people. Various specialists are involved in nutrition support teams, but comprehensive management by family physicians can benefit the care of older patients*.

#### Effectiveness of nutrition support team management by family physicians

Including family physicians in nutrition support teams increased the comprehensiveness of the care provided by the nutrition support team to older patients. The presence of a family physician in the nutrition support team also served as a trigger for noticing various abnormalities. The team members stated that family physicians could comprehend various aspects of the medical conditions of older patients and were therefore suited to direct the team care.

Family Physician A stated,

*Family physicians can notice various changes in medical aspects from different organ symptoms*.” Family Physician C stated, “*Family physicians may be useful for specialized teams such as nutrition support teams. I have learned about various medical fields and cared for many older patients. As a family physician, I can advise a comprehensive way of care*.

Participants recognized the importance of the comprehensive perspective and expertise of the family physician within the nutrition support team in facilitating consensus and approaching diverse patients. Providing comprehensive care enabled the nutrition support team to manage patients from multiple perspectives to improve their nutritional status.

Nutritionist A stated,

*Family physicians have a comprehensive view of older patients and understand multiple medical issues affecting poor nutritional conditions. Such skills can lead the nutrition support team effectively*.

### Comprehensive management through the nutrition support team

#### Comprehensive assessment of patients with acute care-sensitive conditions

The nutrition support team, including the family physician, effectively recognized comprehensive changes in orthopedic patients’ conditions. In addition to nutritional conditions, factors associated with the deterioration of nutritional status were assessed through team discussion. Including family physicians in the team facilitated comprehensive assessment.

Nurse A stated,

*Older patients have multiple medical problems causing various changes in their clinical courses. Focusing on one organ may not effectively assess older patients. Family physicians can consider their complicated situations systematically*.

In addition, a comprehensive assessment enabled subtle changes to be detected in older patients with acute care-sensitive conditions. The clinical condition of older patients with multimorbidity can deteriorate rapidly. Early involvement of the nutrition support team enabled early identification of malnutrition in orthopedic patients and an assessment of the causes.

Family Physician B stated,

*The nutrition team is useful for patients with acute care-sensitive conditions. We can detect their changes at an early stage. Early detection of changes can lead to patient treatment and prevent prolonged hospitalization and wasteful use of healthcare resources*.

#### Systematic approaches to complicated patients through interprofessional collaboration

By actively seeking the opinions of various team members who collaborated with the family physicians, the nutrition support team intervened with patients effectively by consolidating the perspectives of diverse specialists. Participants recornized the importance of diverse perspectives and stated that family medicine was vital for the interventions of older patients with nutritional conditions and multimorbidity.

Nutritionist D stated,

*The nutrition team approaches are not simple and consider various aspects of patients, such as nutritional conditions, medications, and patients’ perceptions regarding their conditions. All of the issues should be discussed to solve the patients’ conditions*.

Family Physician B stated,

*Especially older patients need systematic approaches regarding various factors related to their lives. In family medicine, a patient-centered approach can be used to provide a systematic approach. Effective ways can be found to approach patients nutritional conditions by considering the biopsychosocial aspects*.

Older patients tend to have multiple medical conditions and frailties. The nutrition support team were required to deal with failure to thrive and issues of terminal illness. Approaching these issues was challenging, but various members’ opinions and respecting biopsychosocial aspects through the team discussion mitigated their difficulties in developing a plan for providing nutrition support.

Nutritionist B stated,

*The nutrition support team’s decision can change older patients’ management, such as withdrawal of treatment or starting end-of-life care. I felt that the decision was a little heavy and too responsible. However, respecting the biopsychosocial aspects of patients and discussing the situation as a team can relieve my mental stress and help us to work together as a team*.

## Discussion

This study identified four theories regarding the role of family physicians’ in providing nutritional support to older orthopedic patients. Traditional interpersonal relationships within the healthcare hierarchy can be a hindrance to collaboration among various professions. Healthcare professionals feel a disparity in their occupational role and avoid expressing their opinions to physicians, causing unconscious adherence to the traditional professional hierarchy and frustration about being unable to use their knowledge. In an aging society, many patients require surgery, causing delays in responding to changes in the condition of orthopedic patients because of the lack of time, due to which many orthopedic patients experience a rapid deterioration in their clinical condition. An increasing awareness of changes in patients’ nutritional conditions was observed through the involvement of the nutrition support team. Through the approach of nutrition support teams to patients with diseases of specific organs, there has been increased recognition of abnormalities in the patient’s conditions other than nutrition. The presence of family physicians in the nutrition support team serves as a catalyst for noticing various abnormalities, facilitating the aggregation of opinions, and managing patients with diverse conditions. The nutrition support team, including the family physician, effectively recognizes changes in patients’ overall symptoms by identifying malnutrition in orthopedic patients. By actively seeking the opinions of various team members, the nutrition support team intervenes effectively by consolidating the viewpoints of diverse specialists.

Hierarchical and relational limitations are invariably present in interprofessional collaborations, and this can have a negative effect on the quality of collaboration [19–21]. The findings of the present study show that rural healthcare professionals believed that their previous relationships and hierarchy affected their collaboration and led to inadequate information sharing and discussion about patients’ care, resulting in a deterioration in patients’ conditions. Especially in rural contexts, each healthcare professional plays a diverse role, and each professional may have a robust professional role in their context, making it difficult for them to adapt to a change in their working relationships with other healthcare professionals [22, 23]. Although the need for interprofessional collaboration has been stressed in medical education, there are strong professional hierarchies that hinder interpersonal collaboration in rural settings [24, 25]. Developing interprofessional teams with a mutual ongoing discussion about the roles of different professionals can mitigate barriers to psychological safety and overcome the professional hierarchy [26]. Therefore, the presence of a professional hierarchy should be considered and approached systematically through effective leadership when initiating an interprofessional collaboration.

Including family medicine physicians within the nutrition support team can prevent delayed detection of the need for geriatric care in orthopedic patients. Specialized care has become the norm in modern medicine; thus, comprehensive care may not be provided to older patients [27]. Adequate care of older patients is crucial to preventing acute deterioration in patients with complicated medical conditions [3, 28]. This study revealed that nutrition support teams can detect subtle changes in older patients through nutrition assessment. By adding the family medicine perspective of comprehensive care, the quality of the assessment can be improved, considering various healthcare perspectives in patients with complicated conditions [3, 29]. In addition, family physicians’ communication and leadership skills can be beneficial for negotiating with other healthcare professionals, including specialist physicians such as orthopedic surgeons [30]. The professional hierarchy of other healthcare professionals can limit interventions by specialists in other specialties [21, 31]. This study revealed that other healthcare professionals were hesitant to report their perspectives to physicians, which delayed the detection of acute conditions in older patients. Including family physicians in the team can mitigate the professional hierarchy and lead to earlier intervention in patients’ conditions [32, 33]. Family physicians’ comprehensive management and communication skills can facilitate effective communication with specialist physicians, enabling nutrition support teams to provide comprehensive management of nutritional conditions.

Capitalizing on the effectiveness of family medicine in rural hospitals can be beneficial for the effective care of older patients. Family medicine is a system-specific specialty, and family physicians can work in communities as primary health care leaders [5, 34]. Family physicians work mainly in clinics for the provision of primary care. Additionally, family physicians can drive primary care and collaborate with community healthcare professionals on the prevention of diseases and end-of-life care [35–37]. Family physicians can also effectively manage hospitalized patients [16]. This study reveals that family medicine can provide comprehensive management through nutrition support teams in rural community hospitals. Acknowledgment of the effectiveness of family medicine in rural hospitals can use family medicine effectively in the management of diverse conditions in hospitalized patients through interprofessional collaboration. Comprehensive care of older patients is required both in primary care and hospital care for the sustainability of community care [38, 39]. Family physicians can be specialists in comprehensive care in hospitals and primary care. The present family medicine education system focuses on primary care management in clinics and community healthcare institutions [40]. In aging societies, more older people are admitted to hospitals because of their vulnerability due to multimorbidity. Family physicians should be educated to serve as generalists in community hospitals, provide comprehensive care for smooth and systemic hospital care of older patients through interactions with specialist physicians with different specialties, and sustain home care of older patients through collaboration with community healthcare professionals.

This study has several limitations. First, the findings may lack transferability as this study was performed in one rural community hospital in Japan. Therefore, to overcome this limitation, the researchers clarified the interactive process and effectiveness of implementation in nutrition support teams through iterative data collection and comprehensive descriptions of the context and learning methods. The data collection was also performed in multiple places in the rural hospital. Similar studies should be performed in different cultures and countries to improve the transferability. Another limitation is reliability. To improve reliability, we used iterative data analysis and collected the data over an extended period. Future studies should investigate the reality and concrete processes of family medicine collaboration with multiple professionals in other regions and countries and assess the applicability of the theoretical model used in this study in other settings. Regarding trustworthiness of the research process, obtaining feedback from team members was performed only in the later part of the study, and this was seen as a limitation. However, to promote trustworthiness, two of the authors coded the data transcripts, which enhanced the validity of the study results based on their different perspectives and backgrounds. Furthermore, a third researcher reviewed the process of coding, concepts, and themes to enable triangulation of investigator perspectives for establishing themes.

## Conclusions

This study identified four theoretical concepts regarding family physician’s role in providing nutrition support to older orthopedic patients. By overcoming professional hierarchy and using the knowledge of different healthcare professionals, the rapid deterioration of orthopedic patients’ conditions can be detected early, and nutrition support teams can provide comprehensive management. The presence of family physicians in the nutrition support team can catalyze noticing various abnormalities, facilitating the aggregation of opinions, and supporting patients with diverse health conditions. Family medicine is important in fostering collaboration with various specialist physicians in rural primary care settings and is vital for the effective care of older patients in community hospitals. The role of family medicine should be investigated in other hospital settings to enable the provision of better care for older patients and to drive mutual learning among healthcare professionals.

### Electronic supplementary material

Below is the link to the electronic supplementary material.

Supplementary Material 1

## Data Availability

The datasets used and/or analyzed during the current study may be obtained from the corresponding author upon reasonable request.

## Abbreviations

ADL: Activities of daily living
