# Supplementary material for: Role of family medicine physicians in providing nutrition support to older patients admitted to orthopedics departments: a grounded theory approach
Source: BMC Prim Care. 2024 Apr 19;25:121. doi: 10.1186/s12875-024-02379-4 (PMC11027398; doi:10.1186/s12875-024-02379-4)
Supplement: Supplementary file 1 — Supplementary Material 1 [file 12875_2024_2379_MOESM1_ESM.docx]

**Interview guide**

**Role of family medicine physicians in providing nutrition support to older patients admitted to orthopedics departments**

We would like to interview you to investigate the role of family medicine physicians in providing nutrition support to older orthopedic patients

**The explanation**

-The interview’s duration is about 60 minutes.

-The contents of the interview were used in this research.

-The interview contents are used for research only.

-The content of the interview will be recorded and transcribed verbatim. The recorded data will be discarded after use to protect personal information.

-If you are inconvenienced, you can withdraw anytime and will not suffer any medical disadvantage.

-In the unlikely event you suffer a disadvantage, you will immediately stop participating in the research and not use the data.

-Participants' information is based on national guidelines, including that privacy and human rights are adequately protected. If you have any questions or concerns regarding this request, please get in touch with the following: Unnan City Hospital Community Care Department: +81854-47-7500

**Interview guide**

I will ask the following four questions:

1. What did you think of the involvement of the Family Medicine Department with orthopedic patients through nutrition support teams?
2. What do you consider to be the challenges and benefits of the involvement of the Family Medicine Department in nutrition support teams?
3. Do you have any suggestions on addressing the challenges and benefits of the involvement of the Family Medicine Department?

Ryuichi Ohta

Unnan city hospital
